# The efficacy and safety of corticosteroids in pediatric kidney scar prevention after urinary tract infection: a systematic review and meta-analysis of randomized clinical trials

**DOI:** 10.1007/s00467-023-05922-0

**Published:** 2023-03-21

**Authors:** Nikolaos Gkiourtzis, Agni Glava, Maria Moutafi, Theopisti Vasileiadou, Theodora Delaporta, Panagiota Michou, Nikoleta Printza, Kali Makedou, Despoina Tramma

**Affiliations:** 1 4th Department of Pediatrics, Papageorgiou General Hospital, Aristotle University of Thessaloniki, Thessaloniki, Greece; 2 Pediatric Department, G. Gennimatas General Hospital, Thessaloniki, Greece; 3 1st Department of Pediatrics, Ippokrateio General Hospital, Aristotle University of Thessaloniki, Thessaloniki, Greece; 4 Laboratory of Biochemistry, School of Medicine, AHEPA University Hospital, Aristotle University of Thessaloniki, Thessaloniki, Greece

**Keywords:** Kidney scars, Corticosteroids, Pyelonephritis, Urinary tract infections, Children

## Abstract

**Background:**

Acute pyelonephritis (APN) in pediatric patients may lead to kidney scarring and is one of the main causes of permanent kidney damage. The incidence of kidney scarring after one febrile urinary tract infection (UTI) is reported to range from 2.8 to 15%, with the percentage rising to 28.6% after ≥ 3 febrile UTIs. Corticosteroids may have a role in the reduction of kidney scar formation and urine cytokine levels. The possible benefit of adjuvant corticosteroid administration in the reduction of kidney scar formation in children with APN has been recently examined in randomized controlled trials (RCTs).

**Objectives:**

The aim of this meta-analysis was to provide a summary of the current literature about the efficacy and safety of adjuvant corticosteroid administration in the reduction of kidney scar formation in children with APN.

**Data sources:**

An extensive literature search through major databases (PubMed/MEDLINE and Scopus) was carried out for RCTs from inception until October 12, 2022, investigating the efficacy and safety of adjuvant corticosteroids in preventing kidney scarring in children with APN. A risk ratio with 95% CI was used for dichotomous outcomes.

**Results:**

In total, 5 RCTs with 918 pediatric patients with APN were included in the study. Adjuvant corticosteroid treatment revealed a statistically significant reduction in kidney scarring (95% CI 0.42–0.95, *p* = 0.03), without increasing the risk of adverse events like bacteremia, prolonged hospitalization, or recurrence of UTI.

**Limitations:**

There were limitations regarding sample size (*n* = 498 children), different classes of corticosteroids (methylprednisolone or dexamethasone), different routes of corticosteroid administration (intravenous or oral), and different day courses (3-day or 4-day course).

**Conclusions:**

Adjuvant corticosteroid administration seems to have a beneficial effect on kidney scar reduction in children with APN. Future studies should focus on the evaluation of the efficacy and safety of corticosteroids in kidney scarring reduction after APN to strengthen the results of our study.

**Graphical Abstract:**

**Figure:**
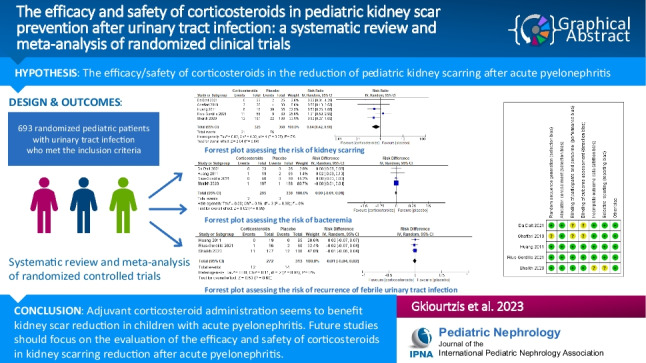
A higher resolution version of the Graphical abstract is available as Supplementary information

**Supplementary Information:**

The online version contains supplementary material available at 10.1007/s00467-023-05922-0.

## Background

Acute pyelonephritis (APN) in pediatric patients may lead to kidney scarring and is listed as one of the important causes of permanent kidney damage [1, 2]. Acquired scarring because of APN seems to be more common in girls and usually is associated with lower-grade vesicoureteral reflux (VUR) and better outcomes [3]. Kidney scarring may lead to hypertension, proteinuria, and the risk of chronic kidney disease increases when high-grade VUR background is present [1, 2, 4, 5]. The incidence of kidney scarring after one febrile urinary tract infection (UTI) is reported to range from 2.8% to 15%, with the percentage rising to 28.6% after three or more febrile UTIs [5, 6]. Risk factors for kidney scarring are multiple APN episodes, high-grade VUR, bacterial virulence, and delay of treatment with antibiotics, especially in infants with non-specific UTI signs [7, 8]. Adequate antibiotic treatment is the most efficient treatment option for UTI, but it may not be sufficient to prevent kidney scarring [9].

Corticosteroids may have a role in reducing kidney scar formation and urine cytokine levels [10]. Cytokines may predict the severity of kidney damage, playing a key role in kidney scarring after APN as they represent the mediators of an inflammatory process in response to an infection [11–14]. A few studies have attempted to examine the hypothesis that corticosteroids may affect cytokine response and decrease kidney damage after APN, with promising results [12, 15]. Recent randomized controlled studies (RCTs) and a meta-analysis demonstrated that a short period of adjuvant corticosteroids may decrease the risk of kidney scar formation after APN [10, 13, 14]. These results and minimal adverse events make adjuvant corticosteroid administration to antibiotics a promising future treatment option for children with pyelonephritis.

We conducted a systematic review and meta-analysis to clarify the role of adjuvant administration of corticosteroids to antibiotic treatment for kidney scar prevention after APN in pediatric patients.

## Methods

### Study registration

We conducted this meta-analysis according to the guidelines of the Preferred Reporting Items for Systematic Reviews and Meta-analyses (PRISMA) and the Cochrane Handbook for Systematic Reviews of Interventions [16, 17]. On October 12, 2022, a prespecified review protocol was registered in OSF (https://osf.io/gw8b3/).

### Search strategy

An extensive literature search through major databases was carried out for RCTs from inception until October 12, 2022, investigating the efficacy and safety of adjuvant corticosteroids in preventing kidney scarring in children with APN. Our search strategy was based on the electronic search by three reviewers (NG, AG, MM) of the available literature in the main medical e-databases (PubMed/MEDLINE and Scopus) (Supplementary Table 1), including relevant terms for kidney scars, pyelonephritis, corticosteroids, and children. Clinicaltrials.com and OSF were screened for additional data. There were no limitations regarding publication year and language. Finally, we screened all the references from the included studies for additional studies.

### Eligibility criteria

The research question was defined using the following criteria [18]: articles were RCTs published in the English language with no limitation on the publication year; pediatric patients with UTI were over two months of age; adjuvant corticosteroid administration to antibiotics in the prevention of kidney scarring and placebo plus antibiotics were administered to the subjects of the intervention and control groups accordingly; the primary outcomes were the incidence of kidney scarring on dimercaptosuccinic acid scan (DMSA scan) after the intervention with corticosteroids in comparison to placebo administration; the secondary outcomes were mean change in clinical, serological, and imaging parameters; non-RCT studies, studies that included bagged urine collection, and studies that involved patients with a previous history of UTI, urinary tract anomalies, kidney failure, kidney scarring, and taking antibiotics before admission were excluded.

### Data collection and extraction

Two authors (AG and MM) independently performed the search of the literature. The records were extracted and imported into a reference management tool (rayan.qcri.org) and duplicates were removed [19]. Then, they independently screened the retrieved studies (title and abstract) according to the inclusion criteria. The eligibility of the remaining studies was assessed independently by full-text screening and in case of disagreements, a third reviewer (NG) made the final decision. Finally, three reviewers (TV, TD, and PM) independently extracted the data of the eligible studies (publication year, study location, identification number, “NCT” number, number of patients in each study, intervention, and patients’ characteristics) into a pre-specified data extraction form. If any study missed data, corresponding authors were contacted to obtain sufficient data.

### Quality assessment

The risk of bias was assessed by two independent-working examiners (NG and PM) using the revised Cochrane risk-of-bias tool (RoB 2.0 version 5.4.1) for randomized trials for each outcome [17, 20]. The RoB tool consists of five domains: randomization process; deviations from intended interventions; missing outcome data; measurement of the outcome; selection of the reported results. Studies were graded as low risk when all domains were classified as “low risk,” “some concerns,” or “high risk” in studies which had one domain classified as “high risk,” or three domains were classified as “some concerns.” In case of any disagreement, a third senior reviewer (DT) made the final decision.

### Outcome measurements

The primary outcome was kidney scarring incidence after the administration of corticosteroids in pediatric patients with APN. Kidney scarring was defined as a photopenic cortical defect with or without loss of volume or contour. Secondary outcomes were mean change in the following parameters: procalcitonin (PCT), erythrocyte sedimentation rate (ESR), C-reactive protein (CRP), creatinine levels, urinary interleukin-6 (UIL-6) and UIL-8. Incidence of VUR, fever duration, kidney damage severity score at the early DMSA (early RDSS), hospitalization duration, risk of bacteremia, ultrasonographic pathologic features in the acute phase, and incidence of kidney scarring on the DMSA scan were also examined. Finally, we evaluated the distribution of a variety of adverse events.

### Statistical analysis

Review manager software 5.4 (RevMan 5.4) was used for statistical analyses [17]. Data from intention-to-treat analyses (ITT) were used when available. Mean values and standard deviations (SD) were used for quantitative data analysis. Qualitative data were analyzed using a 95% confidence interval (95% CI) and risk ratio (RR) or risk difference (RD) when trials with no outcome events in both treatment and control arms were included [21].

Heterogeneity between the studies was assessed using the *I*^2^ test as < 40% may be low, 30–60% as moderate, 50–90% as substantial, and 75–100% as considerable [17]. When *I*^2^ was > 50%, the random effect model was applied. For the analyses, a *p*-value < 0.05 was considered statistically significant.

Finally, subgroup analyses were conducted based on the corticosteroid of use (dexamethasone).

## Results

### Search results

**Fig. 1:**
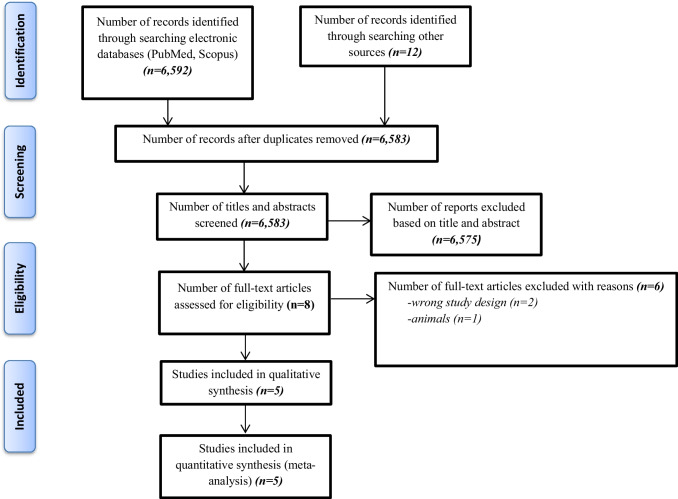
PRISMA flow chart

In total, we identified 6592 records from our initial search. After duplicate removal and title and abstract screening, 8 studies remained for full-text assessment for eligibility, with 5 studies included in the meta-analysis. In total, 693 randomized patients who met the inclusion criteria of the meta-analysis and 498 patients that completed the study (intervention and control groups) were included in the meta-analysis (Fig. 1) [13–15, 22, 23].

### Baseline characteristics

Participants’ mean age ranged from 8.3 (7.9) to 50.55 (44.41) months (Table 1). In four studies, intervention with dexamethasone was made [13, 15, 22, 23] and in only one study [14] methylprednisolone was used as an adjuvant corticosteroid to antibiotic treatment for UTI/APN in pediatric patients. In three studies, the duration of intervention with corticosteroids was for 3 days [14, 22, 23], and in two studies [13, 15], corticosteroids were administered for 4 days. The diagnosis of UTI/APN was made with positive urine culture in three studies [13, 15, 23], and in two studies [14, 22], the diagnosis was made after positive urine culture and DMSA scan evaluation. Finally, in only three studies pediatric patients exclusively with APN were evaluated [14, 15, 22].

**Table 1:** Baseline characteristics of review subjects

Study ID	Trial number	Country	Diagnosis of UTI/APN	Intervention (days)	Antibiotic treatment	Patients with UTI*	Age (months)	Male (%)	Most common UTI bacteria (%)
Da Dalt 2021	EudraCT number: 2013–000,388-10	Italy	Urine culture	Oral dexamethasone 0.15 mg/kg/dose in 2 doses (4 days)	Beta-lactam antibiotics	18	I: 9.0 ± 5.5 P: 8.3 ± 7.9	I: 34% P: 46%	E. coli I: 87% P: 88%
Ghaffari 2019	IRCT20110531006660N4	Iran	Urine culture	Intravenous dexamethasone 0.15 mg/kg/dose in 4 doses (4 days)	Ceftriaxone	52	I: 34.19 ± 30.82 P: 50.55 ± 44.41	I: 8.7% P: 6.9%	N/A
Huang 2011	NCKUH-BR-90–035)	Taiwan	Urine culture and DMSA	Oral methylprednisolone 1.6 mg/kg/day, (max: 48 mg/day) (3 days)	Cephalothin and gentamicin	83	I: 24.6 ± 41.4 P: 20.0 ± 32.4	I: 47.3% P: 52.3%	E. coli I: 89.5% P: 84.6%
Rius-Gordillo 2021	NCT02034851	Spain	Urine culture and DMSA	Intravenous dexamethasone 0.15 mg/kg/dose in 2 doses (3 days)	Amoxicillin-clavulanic, gentamicin or cephalosporin	91	I: 10.3 ± 10.2 P: 11.2 ± 13.4	I: 28% P: 24%	E. coli I: 100% P: 96%
Shaikh 2020	NCT01391793	USA	Urine culture	Oral dexamethasone 0.15 mg/kg/dose in 2 doses (3 days)	Cefdinir	254	I: 73.6% (2–23) 26.4% (24–71) P: 70.2% (2–23) 29.8% (24–71)	I: 7.1% P: 9.0%	E. coli I: 95.1% P: 92.3%

*APN*, acute pyelonephritis; *DMSA*, dimercaptosuccinic acid scan; *E. coli*, *Escherichia coli*; *I*, intervention group; *ID*, identification; *P*, placebo; *UTI*, urinary tract infection; *patients who completed the study

### Risk of bias

**Fig. 2:**
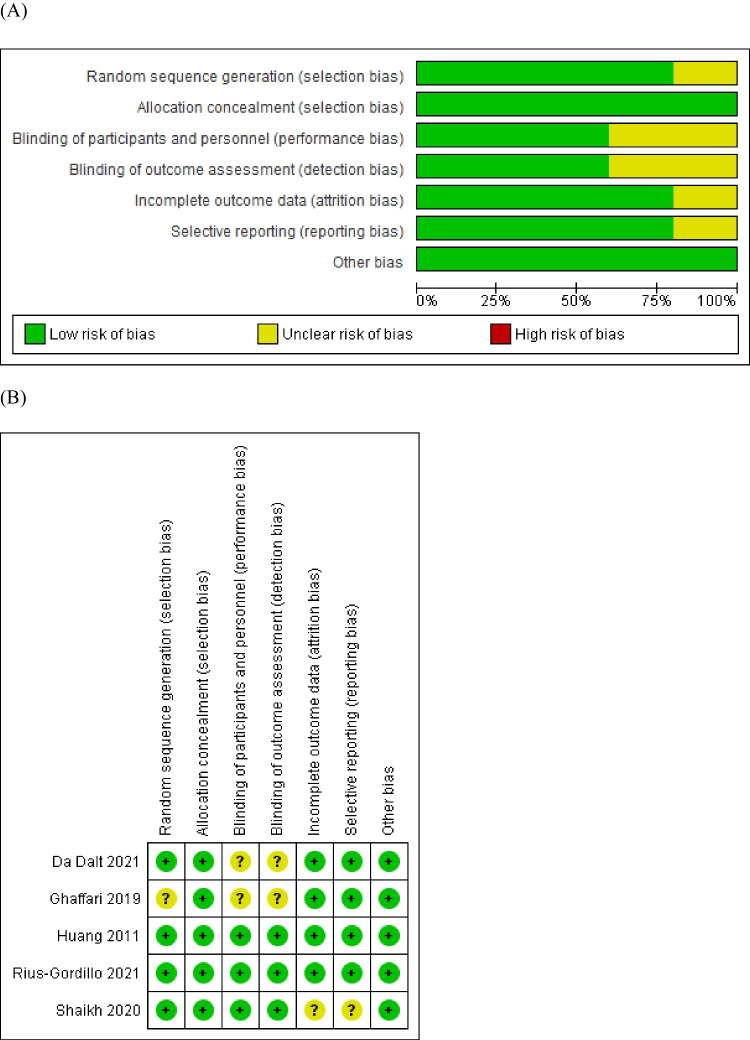
Risk of bias assessment

Four of the five studies included in our meta-analysis were evaluated to be at “low risk of bias” [13, 14, 22, 23]. Only one study was evaluated to be at “some concerns” regarding the lack of well-described blinding processes [15]. A summary of the risk of bias assessment is described in Fig. 2.

### Primary outcome

**Fig. 3:**
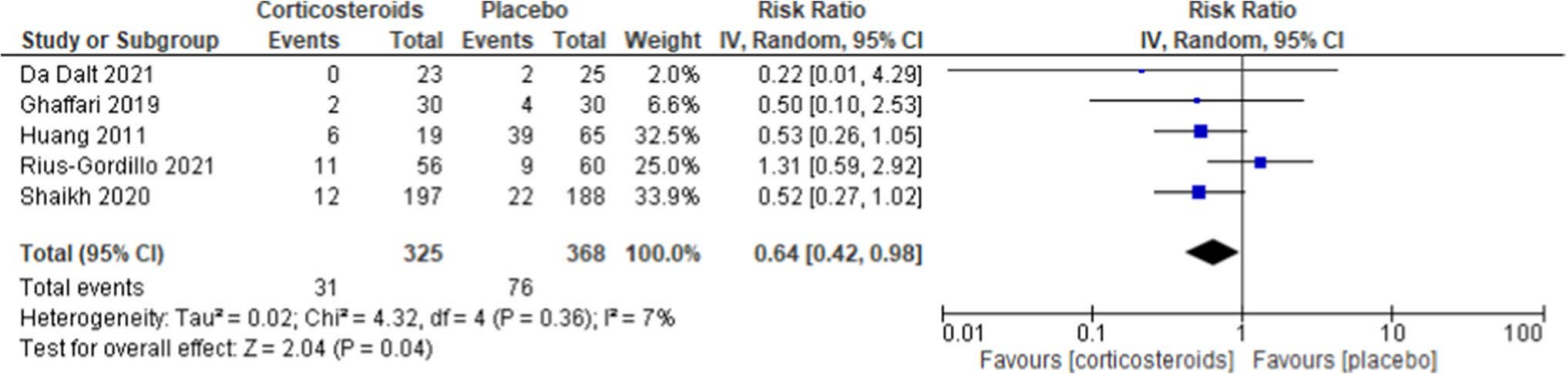
Forest plot assessing the risk of kidney scarring

Co-intervention of corticosteroids with antibiotics showed a significant effect on the incidence of kidney scarring after UTI/APN (RR 0.64, 95% CI 0.42–0.98, *I*^2^ = 7%, *p* = 0.04) (Fig. 3).

### Secondary outcomes

**Fig. 4:**
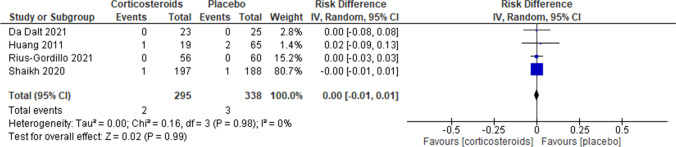
Forest plot assessing the risk of bacteremia

**Fig. 5:**
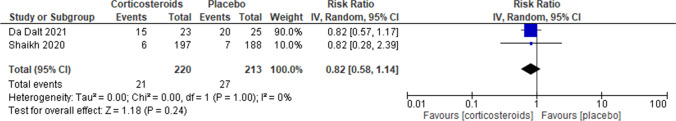
Forest plot assessing the risk of hospitalization

**Fig. 6:**
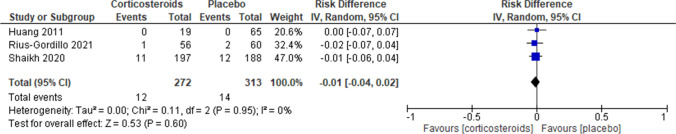
Forest plot assessing the risk of recurrence of febrile. UTI, urinary tract infection

The risk of bacteremia remained the same between the two study groups (RD 0.00, 95% CI –0.01 to 0.01, *I*^2^ = 0%, *p* = 0.99) (Fig. 4). Regarding the length of hospitalization, corticosteroid administration did not lead to any significant change between the two study groups (RR 0.82, 95% CI 0.58–1.14, *I*^2^ = 0%, *p* = 0.24) (Fig. 5). Finally, corticosteroids did not lead to a recurrence of febrile UTI (RD − 0.01, 95% CI − 0.04 to 0.02, *I*^2^ = 0%, *p* = 0.60) (Fig. 6).

As no sufficient data were found for PCT, ESR, CRP, creatine levels, UIL-6/UIL-8, incidence of VUR, fever duration, and early RDSS, we could not come up with a meta-analysis of these endpoints and draw any conclusion.

### Subgroup analysis

#### Dexamethasone administration

Evaluation of the subset of studies that used adjuvant dexamethasone to antibiotics in pediatric patients with APN did not show a significant effect on kidney scarring incidence after UTI/APN (Supplementary Fig. 2).

## Discussion

Our meta-analysis evaluated the effectiveness of adjuvant corticosteroids to adequate antibiotic treatment in the reduction of kidney scar formation after APN/UTI in the pediatric population. The results of our meta-analysis showed that adjuvant corticosteroids to antibiotics led to a statistically significant reduction in kidney scarring incidence after APN/UTI in pediatric patients, without raising the risk of prolonged hospitalization, bacteremia, or recurrence of UTI.

UTI pathogens play a key role in inflammation, with the activation of local and systematic routes after the bacterial invasion [12]. Animal studies have shown that the activation of cytokines during APN can cause damage to the kidney tissue leading to kidney dysfunction [12, 24]. Kidney scarring is the result of the acute inflammation process and although APN is treated with adequate and aggressive antibiotic treatment, there is a high risk of kidney scar formation [12, 25, 26]. Anti-inflammatory agent administration in animal studies has shown statistical significance in the reduction of kidney scarring after APN [25, 27, 28]. Corticosteroids are one of the most used anti-inflammatory agents and the most studied option for kidney scar prevention after APN [29].

A few studies have investigated the effects of corticosteroids for the prevention of kidney scarring after pediatric APN or UTI [10, 12–15, 22, 23]. Meena et al. conducted the first meta-analysis to assess the efficacy and safety of adjuvant corticosteroids for preventing kidney scar formation in children with APN [10]. They included 529 randomized subjects from three RCTs drawing the conclusion that corticosteroids are effective in kidney scarring reduction compared with placebo.

We conducted an extended literature search based on the available literature in the main medical electronic databases (PubMed/MEDLINE and Scopus) with no limitations regarding publication year and language. Our meta-analysis included only well-designed, placebo-controlled RCTs that focused on the pediatric population. Additionally, the analysis was performed with the help of the most recent RoB 2.0 tool and the review procedure was done in accordance with the Cochrane Handbook for Systematic Reviews of Interventions [17, 20]. Moreover, our meta-analysis was characterized by low heterogeneity for all outcomes assessed. Finally, only one study was evaluated to be at “some concerns” with all remaining studies evaluated to be at “low risk of bias” in the quality assessment.

The main advantages of the present systematic review and meta-analysis include the larger population number of included pediatric patients (5 RCTs with 693 randomized patients who met the inclusion criteria of the meta-analysis and 498 patients who completed the study follow-up). We also investigated the effectiveness of corticosteroids based on the corticosteroid of use (dexamethasone).

Our meta-analysis had also some limitations that have to be acknowledged. Corticosteroids used in the RCTs of our analysis do not belong to the same classes, with one study [14] including methylprednisolone and the other four [13, 15, 22, 23] dexamethasone as the corticosteroid of choice. According to the “Coopman classification,” methylprednisolone belongs to class A and dexamethasone to class B corticosteroids [30]. They were also administered via different routes (intravenous or oral) and for different day courses (3-day or 4-day courses). Other limitations are that the total number of subjects who completed the study is relatively small (*n* = 498 children) and that the diagnosis of APN was not confirmed with DMSA in all RCTs, and therefore in three of them [13, 15, 23], the UTI episodes cannot be recorded with certainty as APN. Ghaffari et al. was the only study that evaluated the modification of interleukin levels in the urine, which can be helpful in the estimation of treatment response [15]. Finally, the outcomes of our meta-analysis are limited due to the incompatibility of the possible comparisons between the study outcomes of different RCTs; thus, adverse events and inflammation marker trends before and after co-intervention with corticosteroids could not be thoroughly evaluated.

The subgroup that received dexamethasone did not reach any significant result in kidney scarring reduction. It is believed that this result was influenced by the dynamics of the studies, as that of Huang et al. was the only RCT that led to a significant reduction of kidney scarring after methylprednisolone administration [14]. This study was not included in the subgroup of dexamethasone administration. When all RCTs were included in the meta-analysis*,* Huang et al. received a large weighting (32.5%), influencing the result. In conclusion, differences in corticosteroid classes may have a key role in these results.

As reported by the results of our meta-analysis, corticosteroids—a well-known and routinely used, inexpensive, and relatively safe agent in moderate short-course dosages—could lead to the reduction of the risk of kidney scarring in children with APN, without causing any serious adverse effects. Although there are data that support corticosteroid administration in kidney scarring prevention, current evidence is still insufficient. Further RCTs should evaluate the benefit of corticosteroids in fever duration after their initiation, urinary interleukins, and other serum/urine biomarker levels before and after the intervention and a variety of adverse events.

## Conclusion

In conclusion, adjuvant corticosteroid treatment seems to benefit kidney scar reduction in children with APN. Further well-designed clinical studies examining the efficacy and safety of corticosteroids on kidney scarring reduction after APN should be conducted in the future to strengthen the results of our meta-analysis.

### Supplementary Information

Below is the link to the electronic supplementary material.

Graphical Abstract (PPTX 77 KB)

Supplementary file2 (DOCX 13 KB)

Supplementary file3 (DOCX 21 KB)

Supplementary file4 (DOC 64 KB)
