# Supplementary material for: The efficacy and safety of corticosteroids in pediatric kidney scar prevention after urinary tract infection: a systematic review and meta-analysis of randomized clinical trials
Source: Pediatr Nephrol. 2023 Mar 21;38(12):3937–45. doi: 10.1007/s00467-023-05922-0 (PMC10584697; doi:10.1007/s00467-023-05922-0)
Supplement: Supplementary file 1 — Graphical Abstract (PPTX 77 KB) [file 467_2023_5922_MOESM1_ESM.pptx]

## Slide 1
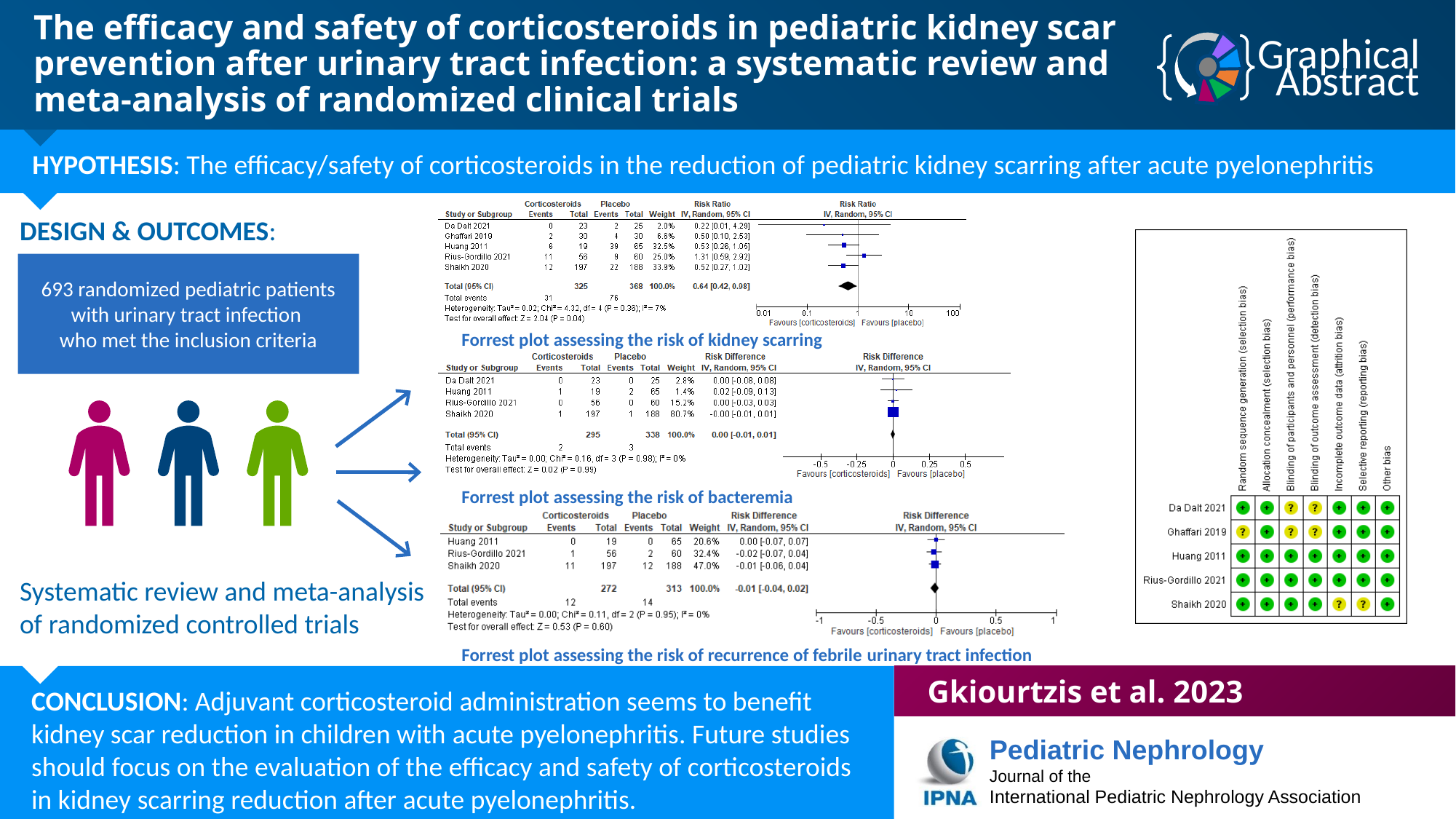

The efficacy and safety of corticosteroids in pediatric kidney scar prevention after urinary tract infection: a systematic review and
meta-analysis of randomized clinical trials
HYPOTHESIS: The efficacy/safety of corticosteroids in the reduction of pediatric kidney scarring after acute pyelonephritis
DESIGN & OUTCOMES:
Systematic review and meta-analysis of randomized controlled trials
693 randomized pediatric patients with urinary tract infection
who met the inclusion criteria
Forrest plot assessing the risk of kidney scarring
Forrest plot assessing the risk of bacteremia
Forrest plot assessing the risk of recurrence of febrile urinary tract infection
Gkiourtzis et al. 2023
CONCLUSION: Adjuvant corticosteroid administration seems to benefit kidney scar reduction in children with acute pyelonephritis. Future studies should focus on the evaluation of the efficacy and safety of corticosteroids in kidney scarring reduction after acute pyelonephritis.
