# Supplementary material for: The efficacy and safety of corticosteroids in pediatric kidney scar prevention after urinary tract infection: a systematic review and meta-analysis of randomized clinical trials
Source: Pediatr Nephrol. 2023 Mar 21;38(12):3937–45. doi: 10.1007/s00467-023-05922-0 (PMC10584697; doi:10.1007/s00467-023-05922-0)
Supplement: Supplementary file 2 — Supplementary file2 (DOCX 13 KB) [file 467_2023_5922_MOESM2_ESM.docx]

| **Number of records identified through searching electronic databases** |
| --- |
| **PubMed/MEDLINE** (05/10/2022) |
| ((((((kidney scars) OR (renal scars)) AND (pyelonephritis)) OR (urinary tract infection)) AND (corticosteroids)) OR (dexamethasone)) AND (children) |
| **Results**: 6,515 |
| **Scopus (**05/10/2022) |
| ( "kidney scars" ) OR ( "renal scars" ) AND ( "pyelonephritis" ) OR ( "urinary tract infection" ) AND ( "corticosteroids" ) OR ( "dexamethasone" ) AND ( "children" ) |
| **Results:** 77 |
