# Supplementary figures and images for: The efficacy and safety of corticosteroids in pediatric kidney scar prevention after urinary tract infection: a systematic review and meta-analysis of randomized clinical trials
Source: Pediatr Nephrol. 2023 Mar 21;38(12):3937–45. doi: 10.1007/s00467-023-05922-0 (PMC10584697; doi:10.1007/s00467-023-05922-0)

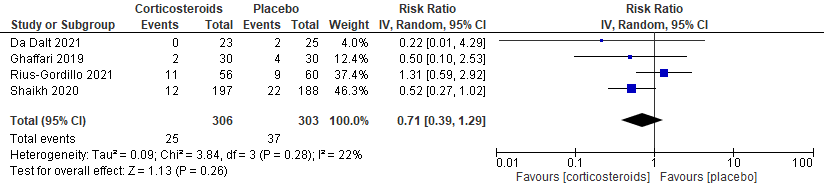

Supplement: Supplementary file 3 — Supplementary file3 (DOCX 21 KB) [file 467_2023_5922_MOESM3_ESM.docx]
